# Study of Interatomic Potentials Using the Crystal-GRID Method on Oriented Single Crystals of *Ni*, *Fe*, and *Cr*

**DOI:** 10.6028/jres.105.009

**Published:** 2000-02-01

**Authors:** N. Stritt, J. Jolie, M. Jentschel, H. G. Börner, C. Doll

**Affiliations:** Institut de Physique, Universitrolles, CH-1700 Fribourg, Switzerland; Institut Laue Langevin, Av. des Martyrs 156X, 38042 Grenoble CEDEX, France

**Keywords:** interatomic potential, lifetime, metals, molecular dynamics simulations, slowing down

## Abstract

The Crystal-GRID method is used to study interatomic collisions at low energy in metals and such to probe the repulsive interatomic potential. Line shapes of gamma rays, emitted by the recoiling ^59^Ni isotope after thermal neutron capture in Ni single crystals, were measured and compared to results obtained by molecular dynamics simulations of the slowing down. The same procedure is also used for recoiling ^57^Fe and ^54^Cr atoms in Fe and Cr single crystals, respectively. Different potentials (including several from the embedded atom method) are investigated using the observed fine structure of the line shape which depends on the crystal orientations. From the detailed study of the lineshapes measured in two different orientations, a new potential is then derived for each element. Nuclear state lifetimes for the excited isotopes are also deduced with a higher precision than obtained with standard nuclear techniques.

## 1. Introduction

The gamma-ray induced doppler broadening (GRID) method [1] is based on the observation of Doppler shifts produced by the motion of radioactive nuclei in solid state targets. The motion of atoms is produced by de-excitation following thermal neutron capture. Usually after this type of reaction, the newly formed isotope will decay by emission of gamma-ray cascades down to the ground state. The GRID technique involves the measurement of the energy of the second gamma ray in a two gamma cascade. The first gamma ray induces a recoil to the nucleus in the opposite direction of emission. If the nucleus emits a second gamma ray while still in flight, the measured energy of the latter will be Doppler shifted. As the gamma rays are emitted isotropically, this results in a Doppler broadened line shape rather than a line that is Doppler shifted. This small Doppler broadening can be measured by the two-axis flat crystals spectrometer GAMS4 [2] installed at the Institut Laue Langevin (ILL) in Grenoble (France) in a ILL/NIST collaboration. Typical values for the recoiling atom energy analyzed with the transition metals are in the order of 200 eV to 400 eV. With this kinetic energy, the atom has enough energy to move from its lattice position and make collisions with its neighboring atoms. Before the excited nuclei emits the second gamma ray (the probability of emission is determined by the nuclear state lifetime), the atom will slow down due to interactions with its neighbors. The slowing down process is simulated by molecular dynamics programs where different interatomic potentials can be implemented. The resulting Doppler line shape is compared to the measured one via a fitting routine [3]. The Crystal-GRID technique [4] uses single crystals as targets, and was developed to study and to construct new interatomic potentials. Here we apply this method to analyze three transition metals: nickel, iron, and chromium.

The Doppler broadened line shape depends on three contributions: i) the lifetime of the nuclear state, which gives the probability of emission of the second gamma ray at a given time, ii) the orientation of the crystal with respect to the direction of observation, which will be discussed in Sec. 2 and iii) the slowing down process, which will be discussed in detail in Sec. 3. The results of the different slowing down theories for the three transitions metals are summarized in Sec. 4. Section 5 deals with the construction of a new potential derived from the measured Crystal-GRID data.

## 2. Crystal-GRID Technique

The gamma ray induced doppler broadening measurements are realized with single crystals as target. The targets consists of three crystals with a dimension of 2 mm × 18 mm × 20 mm having a total mass of 10 g to 15 g. The targets are placed oriented inside the nuclear reactor and are irradiated with a neutron flux of 5 × 10^14^ cm^−2^ s^−1^. The different isotopes and nuclear reaction studied in this experiment are listed below:

$$\begin{array}{l} {}_{28}^{58}{\mathrm{Ni}}_{30}+n\left(25\;\mathrm{meV}\right)\to_{28}^{59}{\mathrm{Ni}}_{31}*\to_{28}^{59}{\mathrm{Ni}}_{31}+\gamma \\ {}_{28}^{56}{\mathrm{Fe}}_{30}+n\left(25\;\mathrm{meV}\right)\to_{26}^{57}{\mathrm{Fe}}_{31}*\to_{26}^{57}{\mathrm{Fe}}_{31}+\gamma \\ {}_{24}^{53}{\mathrm{Cr}}_{29}+n\left(25\;\mathrm{meV}\right)\to_{24}^{54}{\mathrm{Cr}}_{30}*\to_{24}^{54}{\mathrm{Cr}}_{30}+\gamma \end{array}$$ (1)

**Fig. 1:**
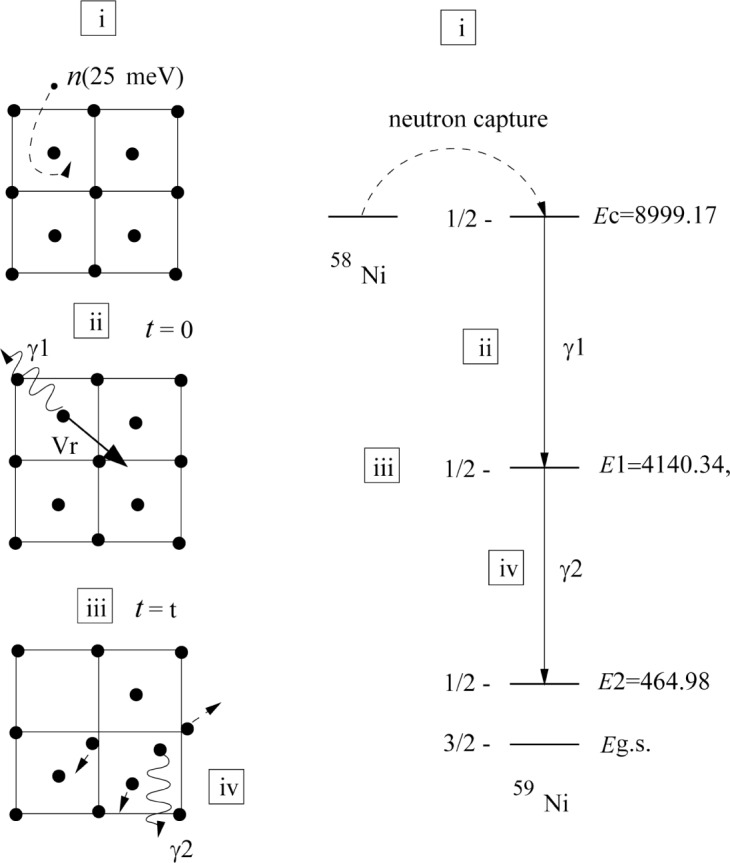
Description of the nuclear reaction and the nuclear state levels and transitions of interest for the Ni isotope (right) and the associated atomic events (left). For simplicity, the motion of the recoiling atom in the crystal is restricted in one dimension.

The process involved in the production of a Doppler broadened gamma-ray line shape are described in Fig. 1. First (i), neutron capture forms an excited nucleus. The newly formed atom will deexcite by emitting a first gamma ray (ii). This emission will induce a recoil to the nucleus and the atom starts to move in the crystal (iii). If a second gamma ray is emitted during the flight (iv) at a time depending on the nuclear state lifetime (*τ*), it will be Doppler shifted due to the emitting atom velocity. The GAMS4 spectrometer measures the energy of the second gamma ray with respect to a fixed direction and records this Doppler shift leading to the line shape. If the recoil is induced by only one primary gamma ray (no cascade or side feeding) then the kinetic energy (*E*_r_) of the atom at the beginning of the recoil is given by:

$$E_{r}=E_{\gamma 1}{}^{2}/2Mc^{2}$$ (2)

where *E*_γ1_ is the energy of the first gamma ray emitted and *M* is the mass of the recoiling atom. Table 1 shows the nuclear levels as well as the different transitions characteristics investigated during this analysis. All of the nuclear levels used are primary almost 100 % directly fed, which gives rise to a unique recoil velocity. As the nuclear state lifetime can be known by other means (DSAM, etc.), and as the direction of observation with respect to the orientation of the crystal is fixed, the data analysis gives valuable information on the slowing down process.

Specially adapted molecular dynamics (MD) programs [5] calculate the trajectories and velocities of the recoiling atom depending on the interatomic potential by solving the equations of motion of a set of atoms. Once the trajectories of the recoiling atoms have been simulated, the only free parameter left over, which can be varied during the fitting procedure, is the lifetime of the nuclear state. If the lifetime is very short, the atom will have a great velocity when it emits the second gamma ray as no collision has taken placed and the atom did not have sufficient time to slow down. Therefore the Doppler line shape will be more broadened.

**Fig. 2:**
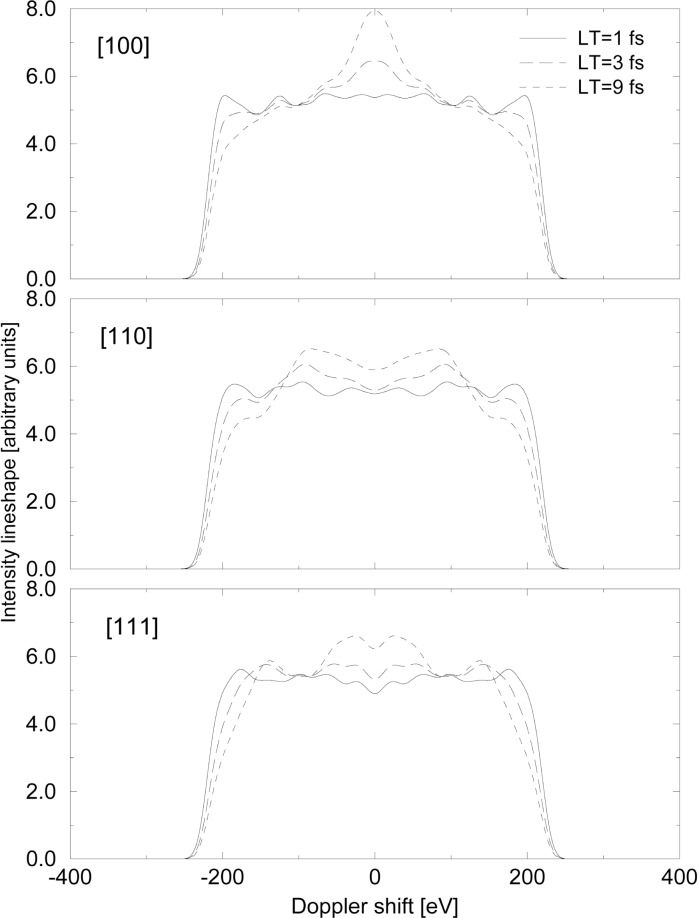
Simulated line shape obtained with the ZBL potential for different lifetime values and different crystal orientations for Fe.

To reconstruct the line shape from the trajectories and velocities given by the MD simulation, the scalar product between the velocity and the direction of observation (given by the direction of the spectrometer) is calculated. This gives the Doppler shifted energy of the emitted gamma ray. Then a summation over all trajectories is realized weighted by the number of gamma rays emitted at that particular time. The probability of gamma emission is given by the exponential decay law assuming a lifetime value. Figure 2 shows the simulated line shape obtained for different lifetime values and different crystal orientations.

Once the potential has been chosen, the trajectories and velocities of the recoiling atoms are stored, the only free parameter is the lifetime. This value is optimized in order to reproduce the measured Doppler broadened line shape. This fitting procedure returns a value for the lifetime and a *χ*^2^ per degree of freedom which tells about the agreement/disagreement between the measured and simulated line shape. A perfect match between the two line shapes will result in a *χ*^2^ equal to one.

**Fig. 3:**
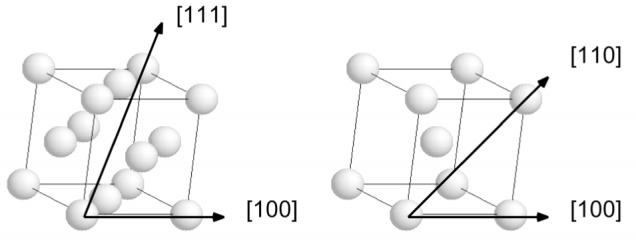
Direction of observation for the two crystalline structures. Ni has a fcc structure shown on the left with the two orientations measured and Fe and Cr have a bcc crystalline structure.

The crystal orientation influences the Doppler broadened line structure as blocking and channeling of the recoiling atom due to the ordered structure of the atoms in the target influences the trajectories and the velocities of the recoiling atoms. Therefore the line shape will show different patterns for different orientations. Ni, Fe, and Cr have simple fcc or bcc crystalline structure. Two orientations for each transitions metals were investigated, and are illustrated in Fig. 3. These two orientations were chosen based on the crystalline structure, because they should show the biggest variation in the Doppler broadened line shape, as the distance separating two closest neighbors is quite different for these orientations.

## 3. Interatomic Potentials

As the line structure and the fitted lifetime depend strongly on how the atom moves inside the crystal, the interatomic potential plays a great role in the data analysis. There exists in the literature many interatomic potentials for metals and many ways to elaborate them. They are usually derived from bulk properties and fitted to experimental data when measurable. Many parametrised forms and formulas (exponential, polynomes, spline knots, etc.) were developed in order to reproduce the value of the pair potential as a function of interatomic distance.

**Fig. 4:**
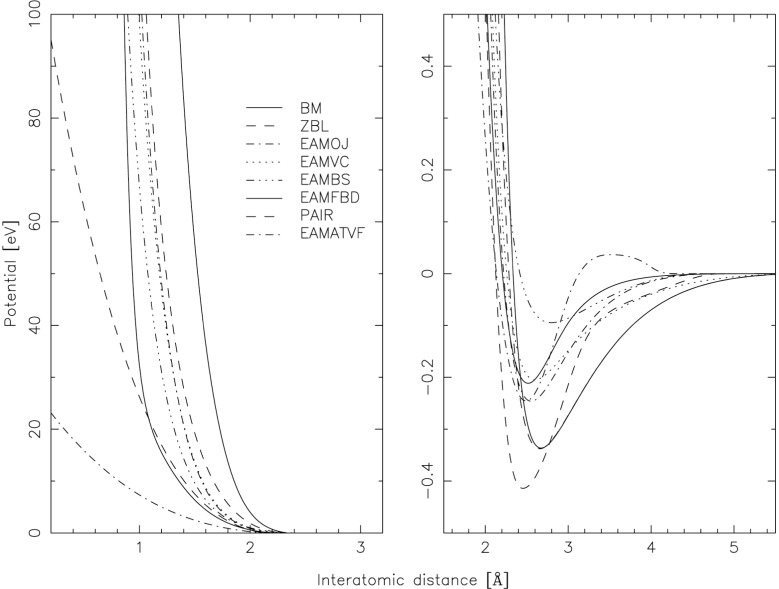
Theoretical interatomic potentials for the Ni-Ni interaction. All the potentials are placed in the figure from the most (BM) to the least (EAMATVF) repulsive potential. For the name of the potentials refer to Table 2.

Table 2 gives a list of some of the potentials used for the slowing down of atoms in metals. Only the best four potentials investigated are given in the table. These potentials are derived so to reproduce different characteristics of bulk properties, such as cohesive energy, lattice constant, elastic constants, properties of stable structure, etc. All of the mentioned potentials are pair potentials and are only dependent on the interatomic distance, even the recent developed embedded atom method (EAM) potentials [9]. In this new approach developed for metals, the atom is seen as embedded in the electron density caused by the neighboring atom and the total energy of the system can be described by the summation over all atoms of an embedding function evaluated for an electron density and a core repulsive term. An approximation to have the force acting on one atom is needed in order to avoid the double summation (summation over all electron density and summation over all atoms). The approximation replaces the electron density by an average electron density. A Taylor expansion over this average electron density is then realized for the evaluation of the needed parameters [10]. Figure 4 shows the form of the different potentials for the Ni-Ni interaction. More potentials are given in the figure in order to illustrate the wide range of potentials found in the literature. A detailed presentation of all the potentials can be found in Ref. [11].

## 4. Results

Many of the mentioned potentials can reproduce quite well bulk properties as they were designed for this purpose, especially the EAM potentials, however not all of them are suited for the slowing down in the energy range of a few hundreds of eV. The Crystal-GRID technique can make a selection of the best interatomic potential for metals by comparing the lifetime value obtained and the *χ*^2^ per degree of freedom which stands for the agreement/disagreement between the stimulated and measured Doppler broadened line shape. The result for the different slowing down theories are listed in Table 3 for the three transitions metals.

**Fig. 5:**
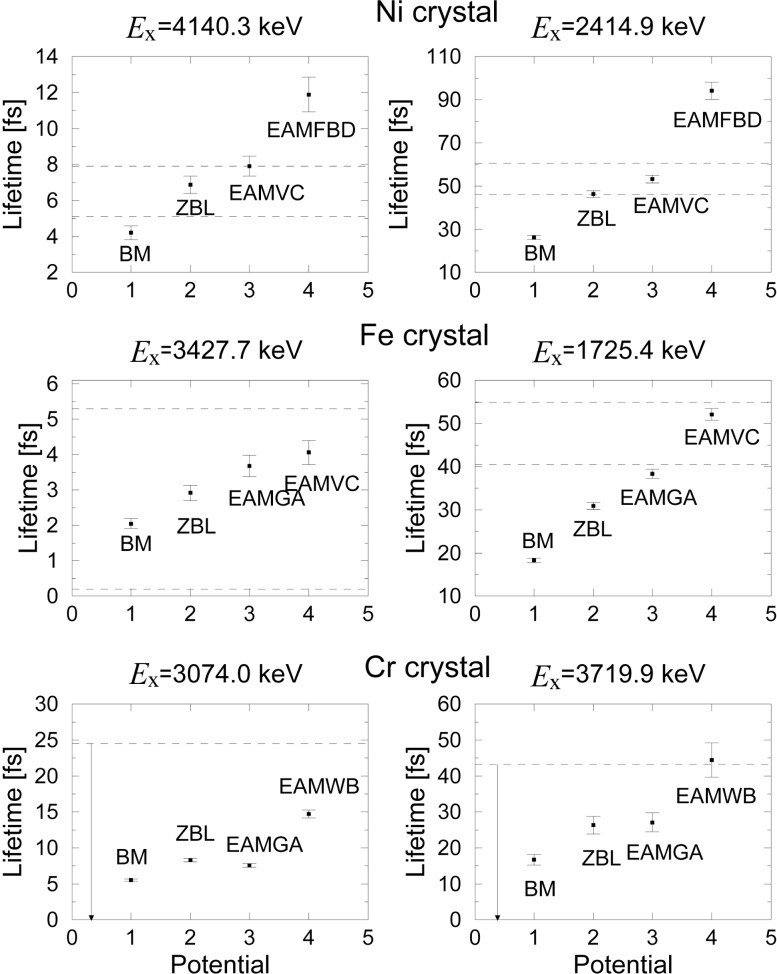
Fitted lifetimes for the three transition metals. Two nuclear state levels were analyzed. The dotted line represents the known lifetime range found in the literature.

For nickel and iron, the best potential, which reproduces the known nuclear lifetime and which gives the smallest *χ*^2^ per degree of freedom, is the embedded atom method potential from Voter and Chen (EAMVC). For chromium, the best candidate is the embedded atom method potential developed by Wang and Boerker (EAMWB). Figure 5 shows the lifetime value obtained with the different potentials and the known lifetime found in the literature for the three transition metals. Among the list of interatomic potentials found, some give lifetime value 3–4 times higher or lower than the known lifetime. This shows that these potentials are not suited in this energy domain.

**Fig. 6:**
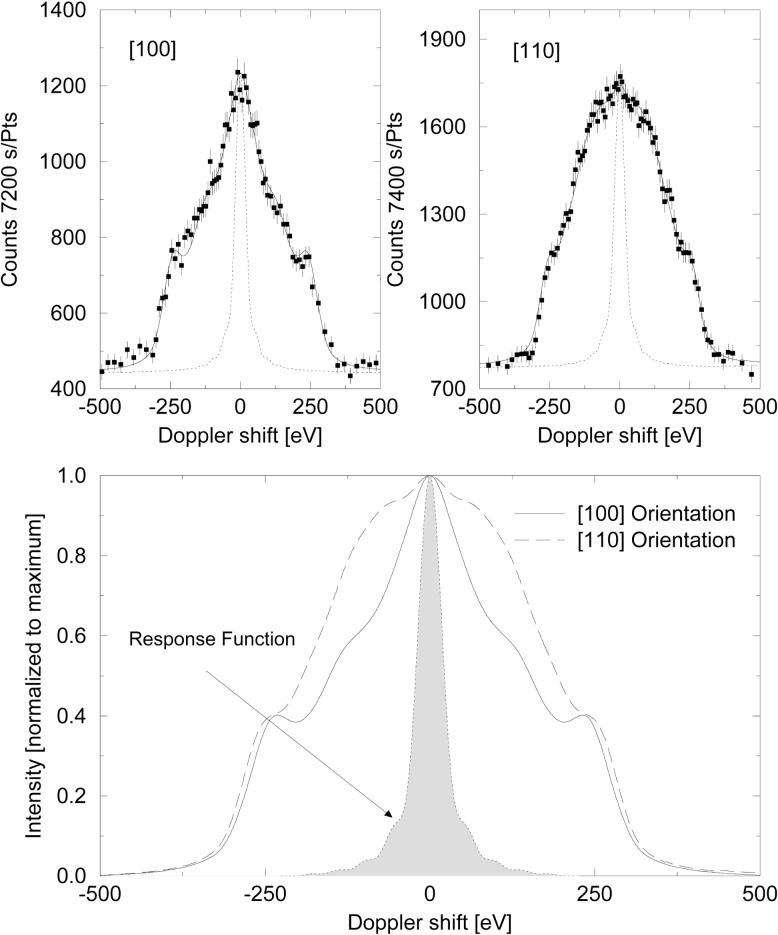
The gamma-ray line shape of the 2239.07 keV transition for chromium in the [100] and the [110] orientation. The lower part shows the fitted line shape for both orientations with the maximum intensity normalized to 1.0.

The orientation of the crystal also influences the Doppler broadened line structure. Figure 6 clearly demonstrates that the blocking and channeling due to the ordered atom position in a crystal gives rise to different line profiles. For example, in a bcc crystal (in the case of chromium), the atom is more free to move in the [110] direction, because the closest neighbor is further (see Fig. 3) than in the [100] direction. Therefore, the line profile will be more broadened in this particular direction compared to the [100] orientation profile.

## 5. Construction of a New Potential

**Fig. 7:**
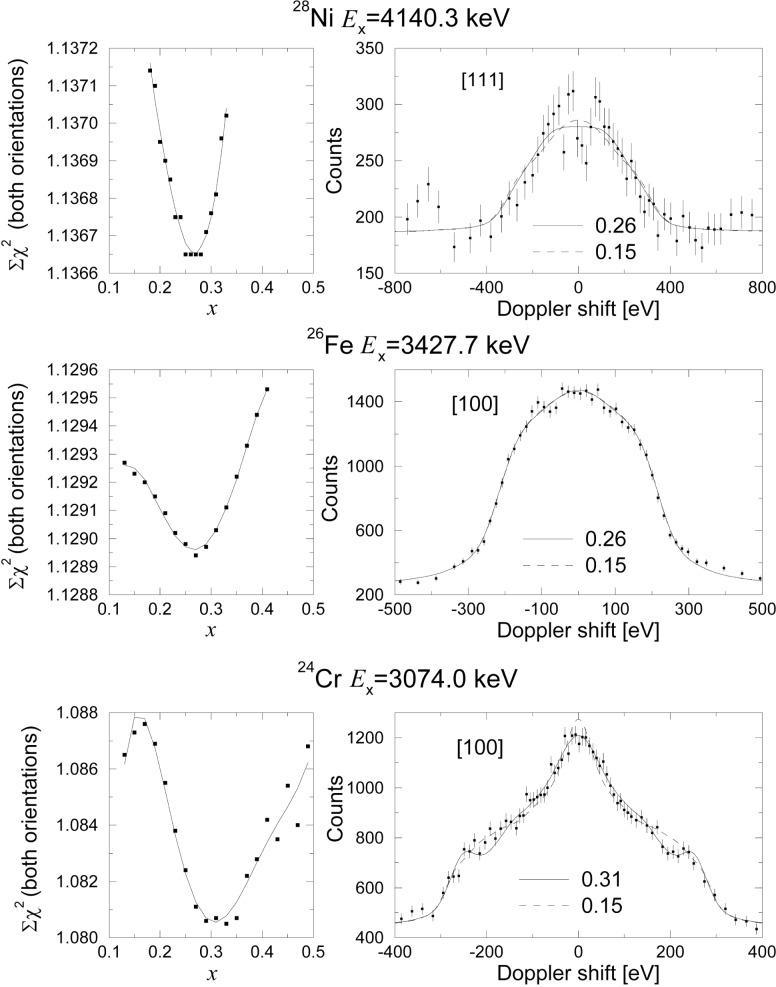
The left shows the variation of the *χ*^2^ per degree of freedom as a function of the *x* parameter for the ZBL potential for Ni, Fe, and Cr. The minimum of the *χ*^2^ is obtained by a 5th order polynomial fit (full line). The right plot represents the fitted Doppler broadened lineshape for the best *x* value (full line) and for an *x* value of 0.15 (dotted line).

From the GAMS4 data a new potential can be derived. This potential is based on the known potential given by Ziegler et al. This ZBL potential is a Coulomb screened pair potential with several fixed parameters. The potential is given by

$$V\left(r_{ij}\right)=\frac{e^{2}}{4\pi \varepsilon_{0}}\cdot \frac{Z_{i}Z_{j}}{r_{ij}}\Phi \left(r_{ij}\right)$$ (3)

where the screening function *Φ* has the form

$$\Phi \left(r_{ij}\right)=\sum_{l=1}^{4}c_{l}\;\exp\left(\frac{d_{l}\;r_{ij}}{a_{s}}\right)$$ (4)

and the screening length *a*_s_ is defined by

$$a_{s}=0.8853a_{B}{\left(Z_{i}^{x}+Z_{j}^{x}\right)}^{y}$$ (5)

with *a*_B_ = 0.529 Å the Bohr radius and *Z_i,j_* the atomic number of the atoms *i* and *j* respectively. All the parameters (*c_l_*, *d_l_*, *x*, *y*) for the evaluation of the ZBL potential are listed in Ref. [12]. As we are dealing with atoms recoiling in the same material, the *x* and *y* parameters can be considered to be one unique parameter. For the construction of the new potential, all the parameters are kept identical, except the *x* parameter, which is varied from 0.05 to 0.40. The original *x* value is equal to 0.23 [9]. An optimization of the *x* parameter is realized by finding the minimum for the *χ*^2^ per degree of freedom obtained by comparing the simulated with the measured line shape. Figure 7 shows the behavior of the *χ*^2^ as a function of the *x* parameter for the three transition metals. The best *x* value for the nickel and iron crystal is equal to 0.26 and for chromium to 0.31. The new derived potential has a form similar to the best slowing down theories found from the analysis when the selection was realized with the lifetime and *χ*^2^ per degree of freedom as criteria. The construction of the new potential from the Crystal-GRID measurement is independent of the nuclear state lifetime which in some case is not known with sufficient precision by other methods.

## 6. Conclusion

We have investigated the interatomic potentials for three transition metals using the Crystal-GRID technique. This new method allows one to make a selection of the best slowing down description among a set of interatomic potentials and also permits to construct new potentials. Two criteria are preponderant for this analysis: i) the nuclear state lifetime and the *χ*^2^ per degree of freedom which is an indication of the agreement or disagreement between the simulated and measured line shape. Thus of a multitude of interatomic potentials for metals, the Crystal-GRID method shows that not all of them are valuable in the energy domain up to a few hundreds of eV. For this particular energy domain, where very few experiments exist, the Crystal-GRID method gives an opportunity to test the veracity of the slowing down of atoms in metals. This nuclear technique based on the observation of Doppler broadening resulting from gamma emission after neutron capture also gives lifetime value for 6 nuclear levels with a much higher precision than standard nuclear techniques.

## Figures and Tables

**Table 1:** The reaction and levels used for the three transitions metals

Reaction	*σ* (barn)	*A* (%)	*E_x_*^a^ (keV)	*E_γ_*^a^ (keV)	*I_γ_*^a^ (%)	∑*I*_in_/∑*I*_out_^a^ (%)	*τ*^a^ (fs)
^58^Ni(n, *γ*)^59^Ni	4.6	68.3	4140.34	3675.23	0.96	100	6.5 (1.4)
			2414.97	1950.05	1.7	96	53.4 (7.2)
^56^Fe(n, *γ*)^57^Fe	2.63	91.7	3427.67	2721.17	1.37	100	4.33 (0.87)
			1725.38	1725.29	6.3	86	47.6 (7.2)
^53^Cr(n, *γ*)^54^Cr	18.2	9.5	3074.00	2239.07	13.9	98	< 25
			3719.75	3719.75	5.04	97	< 43

a Reference [6] for Ni, [7] for Fe, and [8] for Cr.

**Table 2:** Interatomic potential references

Potential name	Authors	Ref.
Ni interaction		
BM	Born Mayer	[12]
ZBL	Ziegler, Biersack, Littmark	[13]
EAMVC	Voter and Chen	[14]
EAMFBD	Foiles, Baskes, Daw	[15]
Fe interaction		
BM	Born Mayer	[8]
ZBL	Ziegler, Biersack, Littmark	[9]
EAMVC	Voter and Chen	[10]
EAMGA	Guellil and Adams	[16]
Cr interaction		
BM	Born Mayer	[8]
ZBL	Ziegler, Biersack, Littmark	[9]
EAMGA	Guellil and Adams	[12]
EAMWB	Wang and Boerker	[17]

**Table 3:** Lifetime results for different potentials obtained with Ni, Fe, and Cr crystals

	*E_x_* (keV)	Known lifetime (fs)	BM (fs)	ZBL (fs)	EAMFBD (fs)	EAMVC (fs)
Nickel ^59^Ni	4140.3	6.5(1.4)	4.21(0.39)	6.88(0.47)	11.89(0.96)	7.95(0.55)
	2414.9	53.4(7.2)	26.24(0.95)	46.30(1.60)	94.18(4.01)	53.06(1.80)
	*χ*^2^^a^		1.1384	1.1371	1.1374	1.1371
Iron ^57^Fe	3427.7^b^	4.33(0.87)	1.95(0.14)	2.82(0.22)	3.58(0.30)	3.96(0.34)
	1725.4	47.6(7.2)	18.30(0.52)	30.92(0.84)	38.34(1.08)	52.13(1.39)
	*χ*^2^^a^		1.1298	1.1292	1.1301	1.1288
Chromium ^54^Cr	3074.0	< 24.5	5.52(0.15)	8.30(0.24)	7.57(0.24)	14.72(0.53)
	3719.9	< 43.3	16.76(1.49)	26.37(2.43)	27.09(2.62)	44.48(4.77)
	*χ*^2^^a^		1.0854	1.0859	1.0918	1.0817

a *χ*^2^ taken for the shortest nuclear state level.

b Gamma-gamma correlation was neglected in this analysis, despite the fact that it might occurs for this very short lived level, due to a lack of experimental knowledge of the mixing ratios.

## References

[1] Börner HG, Jolie J (1993). J Phys G.

[2] Dewey MS, Kessler EG, Greene GL, Deslattes RD, Börner HG, Jolie J (1989). Nucl Instr Meth A.

[3] Robinson SJ, Jolie J (1992). The Computer Code GRIDDLE.

[4] Jentschel M, Heinig KH, Börner HG, Jolie J, Kessler EG (1991). Nucl Instr Meth B.

[5] Stritt N, Jolie J, Jentschel M, Börner HG, Doll C (1998). Phys Rev B.

[6] Baglin CM (1993). Nucl Data Sheets.

[7] Bhat MR (1992). Nucl Data Sheets.

[8] Stuchberry AE, Morrison I, Kennedy DL, Bolotin HH (1980). Nucl Phys A.

[9] Daw MS, Baskes MI (1993). Phys Rev Lett.

[10] Foiles SM, Baskes MI, Daw MS (1986). Phys Rev B.

[11] Stritt N, Jolie J, Jentschel M, Börner HG, Lehmann H (1999). Phys Rev B.

[12] Abrahamson A (1969). Phys Rev.

[13] Ziegler JF, Biersack JP, Littmark U (1985). The Stopping and Range of Ions in Solids.

[14] Voter AF, Chen SP (1987). Mat Res Soc Symp Proc.

[15] Daw MS, Foiles SM (1987). Phys Rev Lett.

[16] Guellil AM, Adams JB (1992). J Mater Res.

[17] Wang YR, Boerker DB (1995). J Appl Phys.

